# Supplementary material for: Efficacy of Adaptogens in Patients with Long COVID-19: A Randomized, Quadruple-Blind, Placebo-Controlled Trial
Source: Pharmaceuticals (Basel). 2022 Mar 11;15(3):345. doi: 10.3390/ph15030345 (PMC8953947; doi:10.3390/ph15030345)
Supplement: Supplementary file 1 [file pharmaceuticals-15-00345-s001.zip › pharmaceuticals-1619360-supplementary/Supplement S2.pdf]

Relief of severity of the Long-COVID symptoms for two weeks treatment follow up for one week.

Figures S1-S14 show the Severity of the Long COVID symptoms and change from baseline during the period of the treatment and follow up (trough 21 days after randomization)

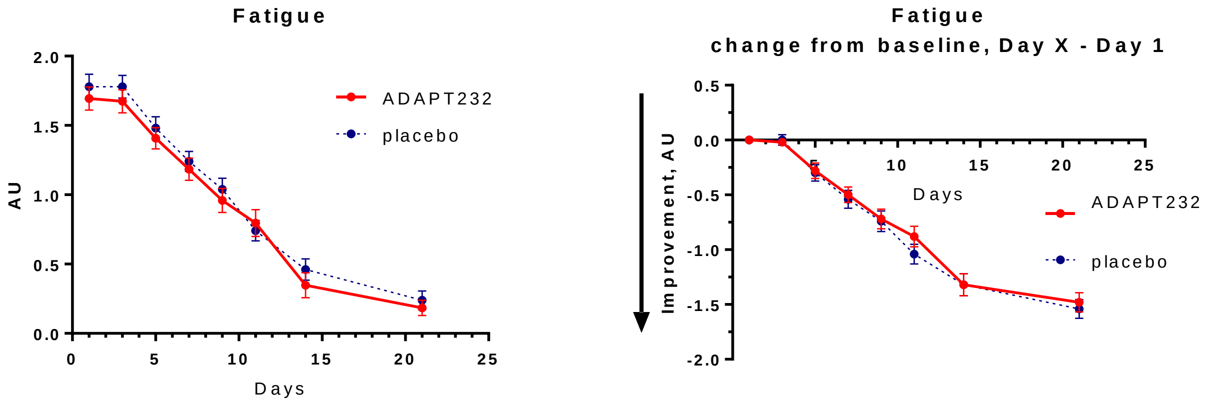

Figure S1. (a) - Fatigue scores (mean ± SEM) of patients in group A (ADAPT-232) and group B (placebo) over the time from Day 1 to Day 21. The changes from the baseline within groups A and B over time were significant (p<0.0001; calculated by Repeated measures ANOVA); two-way ANOVA estimated the significance of the interaction between treatment groups over time; p= 0.9823. The number of patients with symptoms of fatigue in each group is shown in Tables 11 and 12; (b) Between-groups comparison of the changes of fatigue score from the baseline over time shows no interaction (p=0.9664), and no significant difference (p =0.3638) between groups A and B. The ADAPT-232 treatment has no statistically significant effect on fatigue compared to placebo. For details of statistical analysis, see **Supplement 1.**

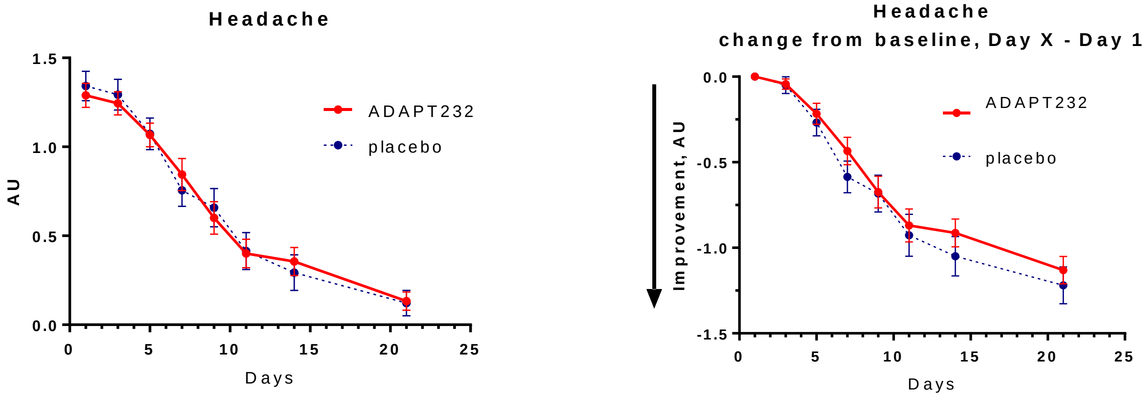

Figure S2. (a) - Headache scores (mean ± SEM) of patients in group A (ADAPT-232) and group B (placebo) over the time from Day 1 to Day 21. The changes from the baseline within groups A and B over time were significant (p<0.0001; calculated by Repeated measures ANOVA); two-way ANOVA estimated the insignificance of the interaction between treatment groups over time; p= 0.983. The number of patients with symptoms of headache in each group is shown in Tables 11 and 12; (b) – Between-groups comparison of the changes of headache score from the baseline over time shows no interaction (p=0.9714), and no significant difference (p= 0.1315) between groups A and B. The ADAPT-232 treatment has no statistically significant effect on headache compared to placebo. For details of statistical analysis, see **Supplement 1.**

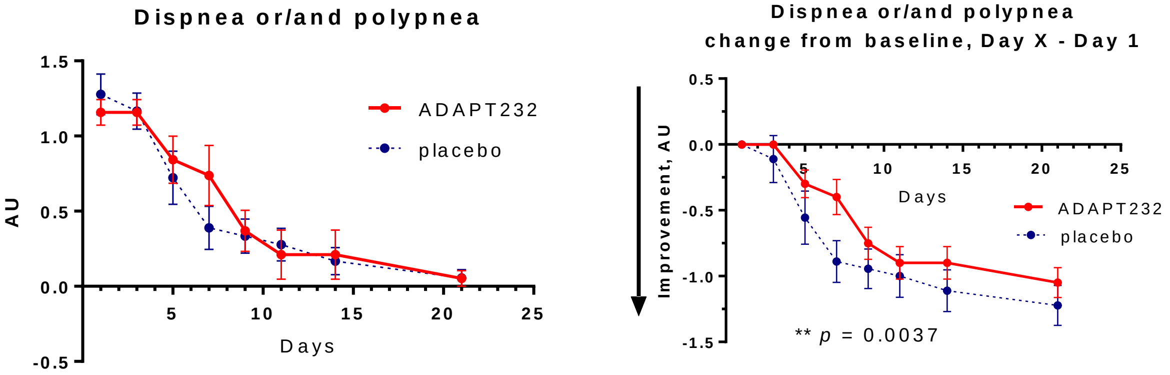

Figure S3. (a) - Respiratory insufficiency scores (mean ± SEM) of patients in group A (ADAPT-232) and group B (placebo) over the time from Day 1 to Day 21. The changes from the baseline within groups A and B over time were significant (p<0.0001; calculated by Repeated measures ANOVA); two-way ANOVA calculated the significance of the interaction between treatment groups over time; p= 0.7679. The number of patients with respiratory insufficiency in each group is shown in Tables 11 and 12. The ADAPT-232 treatment has a statistically significant effect on respiratory insufficiency, decreasing the severity of dyspnea and/or polypnea compared to placebo. (b) Between-groups comparison of the changes of respiratory insufficiency score from the baseline shows no interaction (p = 0.7556) over time but shows a significant difference (p =0.0037) between groups A and B. For details of statistical analysis, see **Supplement 1.**

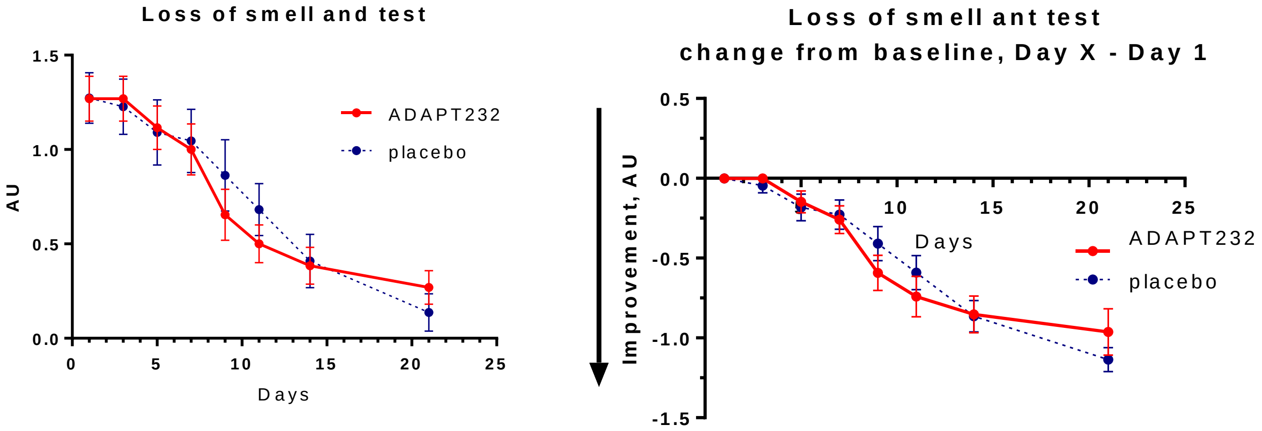

Figure S4. (a) - Organoleptic dysfunctions scores (mean ± SEM) of patients in group A (ADAPT-232) and group B (placebo) over the time from Day 1 to Day 21. The changes from the baseline within groups A and B over time were significant (p<0.0001; calculated by Repeated measures ANOVA); two-way ANOVA calculated the significance of the interaction between treatment groups over time; p= 0.9180. The number of patients with symptoms of Organoleptic dysfunctions in each group is shown in Tables 11 and 12.

(b) - Between-groups comparison of the changes of Organoleptic dysfunctions score from the baseline over time shows no interaction (p= 0.6400), and no significant difference (p = 0.7863) between groups A and B. The ADAPT-232 treatment has no statistically significant effect on loss of smell compared to placebo. For details of statistical analysis, see **Supplement 1.**

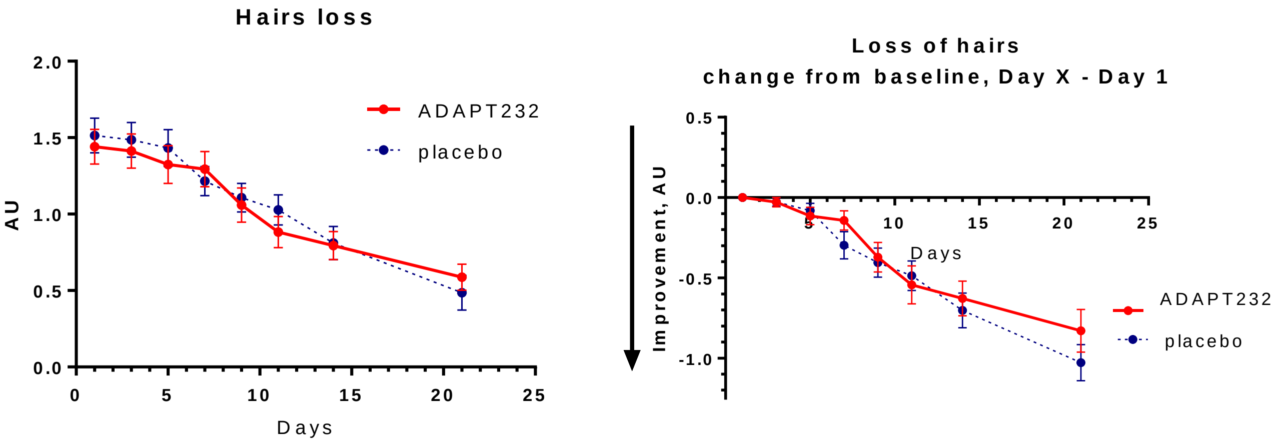

Figure S6. (a) - Hair loss severity scores (mean ± SEM) of patients in group A (ADAPT-232) and group B (placebo) over the time from Day 1 to Day 21. The changes from the baseline within groups A and B over time were significant (p<0.0001; calculated by Repeated measures ANOVA); two-way ANOVA calculated the significance of the interaction between treatment groups over time; p= 0.9441. The number of hair loss symptoms in each group is shown in Tables 11 and 12. (b) - Between-groups comparison of the changes of Hair loss from the baseline over time shows no interaction (p=0.7653), and no significant difference (p = 0.2672) between groups A and B. The ADAPT-232 treatment has no statistically significant effect on loss of hairs compared to placebo. For details of statistical analysis, see **Supplement 1.**

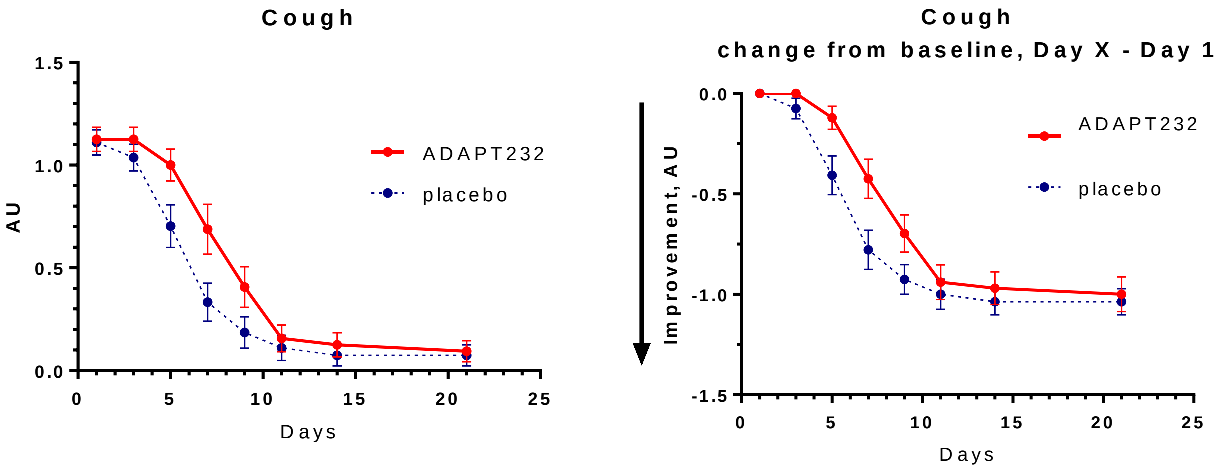

Figure S7. (a) – Cough severity scores (mean ± SEM) of patients in group A (ADAPT-232) and group B (placebo) over the time from Day 1 to Day 21. The changes from the baseline within groups A and B over time were significant (p<0.0001; calculated by Repeated measures ANOVA); two-way ANOVA determined the significance of the interaction between treatment groups over time; p= 0.1466. The number of patients with symptoms of Cough in each group is shown in Tables 11 and 12; (b) - Between-groups comparison of the changes of Cough severity score from the baseline shows interaction (p=0.0993) over time, and significant difference (p = 0.0001) between groups A and B. The ADAPT-232 treatment has a statistically significant effect on cough compared to placebo. For details of statistical analysis, see **Supplement 1**

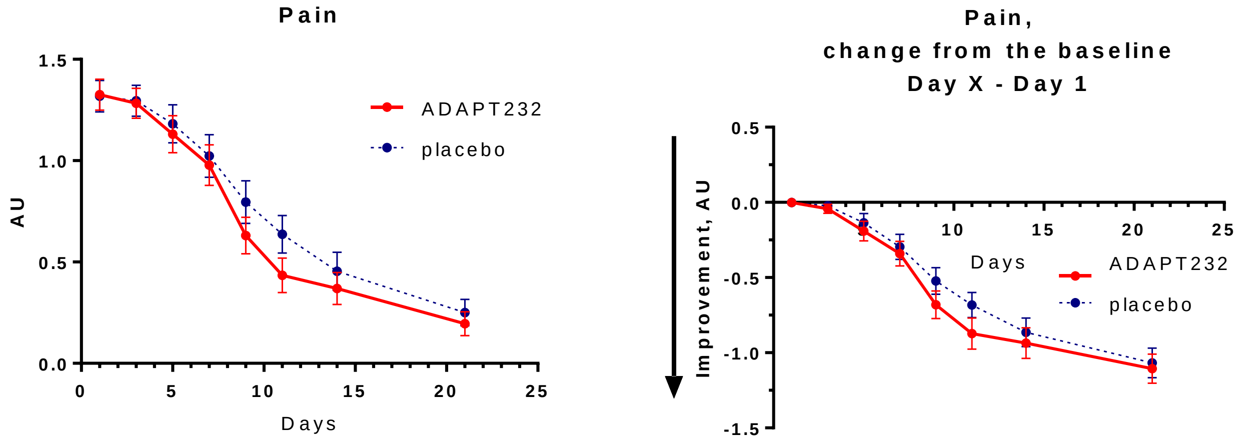

Figure S8. (a) - Pain in muscles, chest, and joints severity scores (mean ± SEM) of patients in group A (ADAPT-232) and group B (placebo) over the time from Day 1 to Day 21. The changes from the baseline within groups A and B over time were significant (*p*<0.0001; calculated by Repeated measures ANOVA). Two-way ANOVA calculated the significance of the interaction between treatment groups over time; the number of patients with pain in muscles, chest, and joints in each group is shown in Tables 11 and 12.

(b) - Between-groups comparison of the changes of Pain score from the baseline over time shows no interaction (p=0.9664), and no significant difference (p =0.3638) between groups A and B. The ADAPT-232 treatment has no statistically significant effect on pain compared to placebo. For details of statistical analysis, see **Supplement 2.**

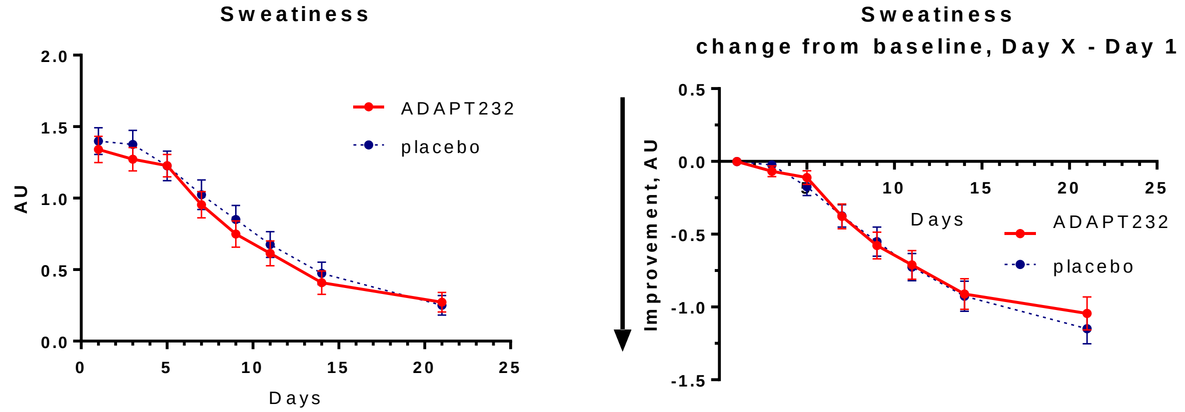

Figure S9. (a) - Sweatiness severity scores (mean ± SEM) of patients in group A (ADAPT-232) and group B (placebo) over the time from Day 1 to Day 21. The changes from the baseline within groups A and B over time were significant (*p*<0.0001; calculated by Repeated measures ANOVA); two-way ANOVA calculated the significance of the interaction between treatment groups over time; *p*= 0.9964. The number of patients with Sweatiness symptoms in each group is shown in **Tables** 11 and 12.

(b) - Between-groups comparison of the changes of Sweatiness score from the baseline over time shows no interaction (p= 0.9903), and no significant difference (*p* = 0.7009) between groups A and B. The ADAPT-232 treatment has no statistically significant effect on sweating compared to placebo. For details of statistical analysis, see **Supplement 2.**

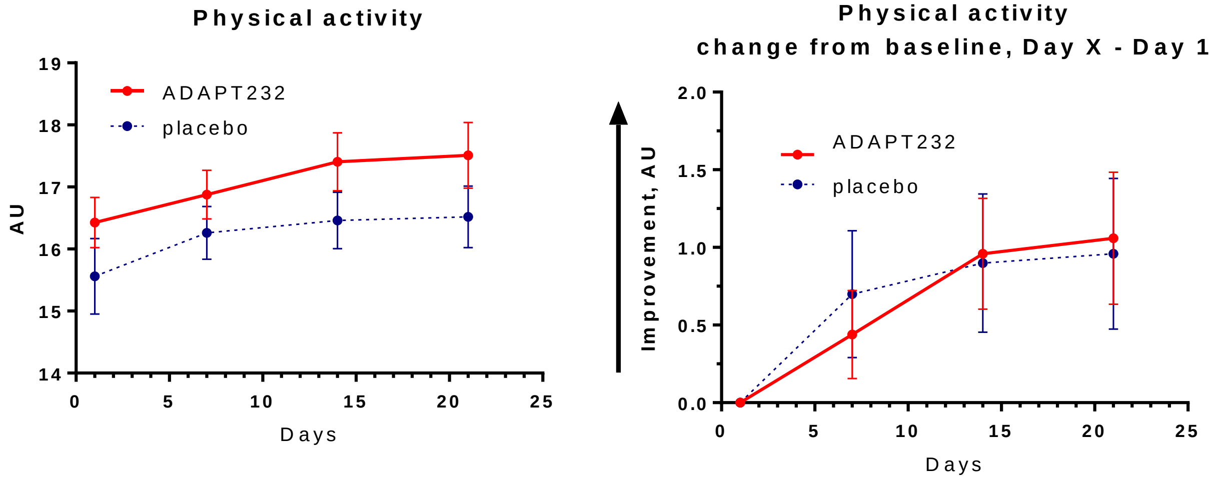

Figure S10. (a) – Physical activity scores (mean ± SEM) of patients in group A (ADAPT-232) and group B (placebo) over the time from Day 1 to Day 21. The changes from the baseline within groups A and B over time were significant (*p*<0.0001; calculated by Repeated measures ANOVA); two-way ANOVA calculated the significance of the interaction between treatment groups over time; *p*= 0.9802. (b) - Between-groups comparison of the changes of Physical activity scores from the baseline over time shows no interaction (p= 0.9569), and no significant difference (p = 0.9203 between groups A and B. The ADAPT-232 treatment has no statistically significant effect on physical activity compared to placebo. For details of statistical analysis, see **Supplement 1.**

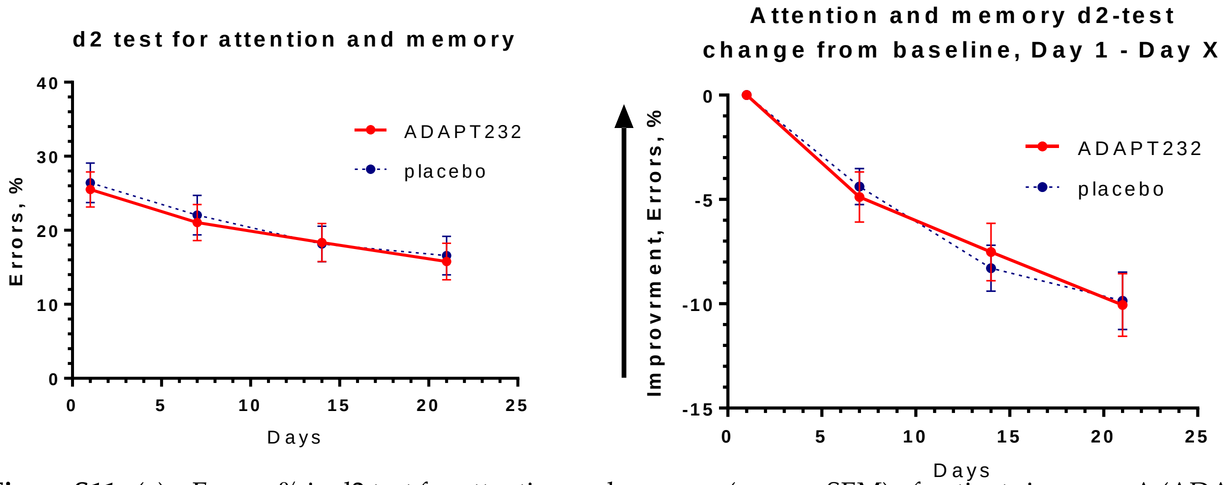

Figure S11. (a) – Errors, % in d2 test for attention and memory (mean ± SEM) of patients in group A (ADAPT-232) and group B (placebo) over the time from Day 1 to Day 21. The changes from the baseline within groups A and B over time were significant (*p*<0.0001; calculated by Repeated measures ANOVA); two-way ANOVA analysis showed no significant interaction between groups over time; *p*= 0.9946. The number of patients included in the analysis is shown in Tables 11 and 12; (b) Between-groups comparison of the changes of Errors, % from the baseline over time shows no interaction (p= 0.9467), and no significant difference (p=0.9816) between groups A and B. The ADAPT-232 treatment has no statistically significant effect on cognitive functions of patients compared to placebo. For details of statistical analysis, see **Supplement 2.**

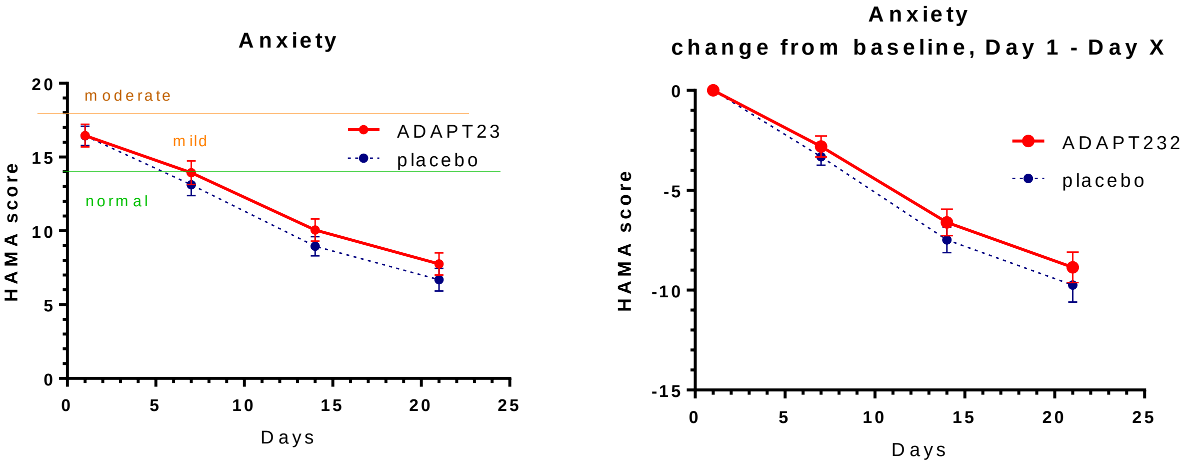

Figure S12. (a) – Anxiety severity scores (mean ± SEM) of patients in group A (ADAPT-232) and group B (placebo) over the time from Day 1 to Day 21. HAMA: 14-17 = Mild Anxiety, 18-24 = Moderate Anxiety, 25-30 = Severe Anxiety. The changes from the baseline within groups A and B over time were significant (p<0.0001; calculated by Repeated measures ANOVA); Two-way ANOVA calculated the significance of the interaction between treatment groups over time; p= 0.8735. The number of patients included in the analysis is shown in Tables 11 and 12; (b) Between-groups comparison of the changes of HAMA score from the baseline over time shows no interaction (p= 0.8528), and no significant difference (p = 0.1609) between groups A and B. The ADAPT-232 treatment has no statistically significant effect on the anxiety of patients compared to placebo. For details of statistical analysis, see **Supplement 1.**

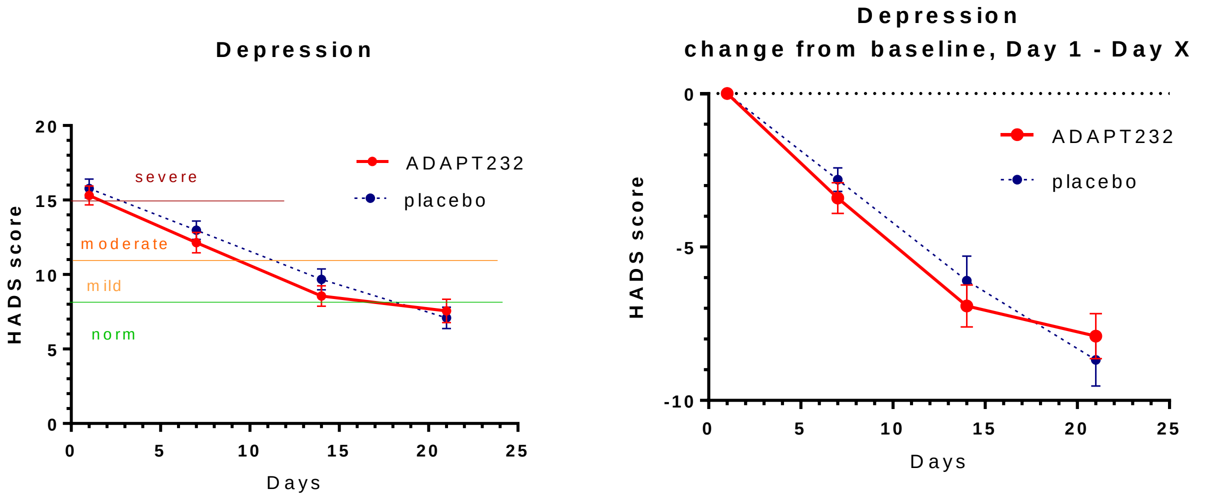

Figure S13. (a) - Depression severity scores (mean ± SEM) of patients in group A (ADAPT-232) and group B (placebo) over the time from Day 1 to Day 21. HADS: normal 0–7, mild 8–10, moderate 11–14, and severe 15–21 anxiety and depression subscales. The changes from the baseline within groups A and B over time were significant (p<0.0001; calculated by Repeated measures ANOVA); two-way ANOVA calculated the significance of the interaction between groups over time; p= 0.6839. The number of patients included in the analysis is shown in Tables 11 and 12; (b) Between-groups comparison of the changes of HADS from the baseline over time shows no interaction (p= 0.5314), and no significant difference (p = 0.7021) between groups A and B. The ADAPT-232 treatment has no statistically significant effect on depression of patients compared to placebo. For details of statistical analysis, see **Supplement 1**.

Duration of Long COVID symptoms over the time of treatment and follow up

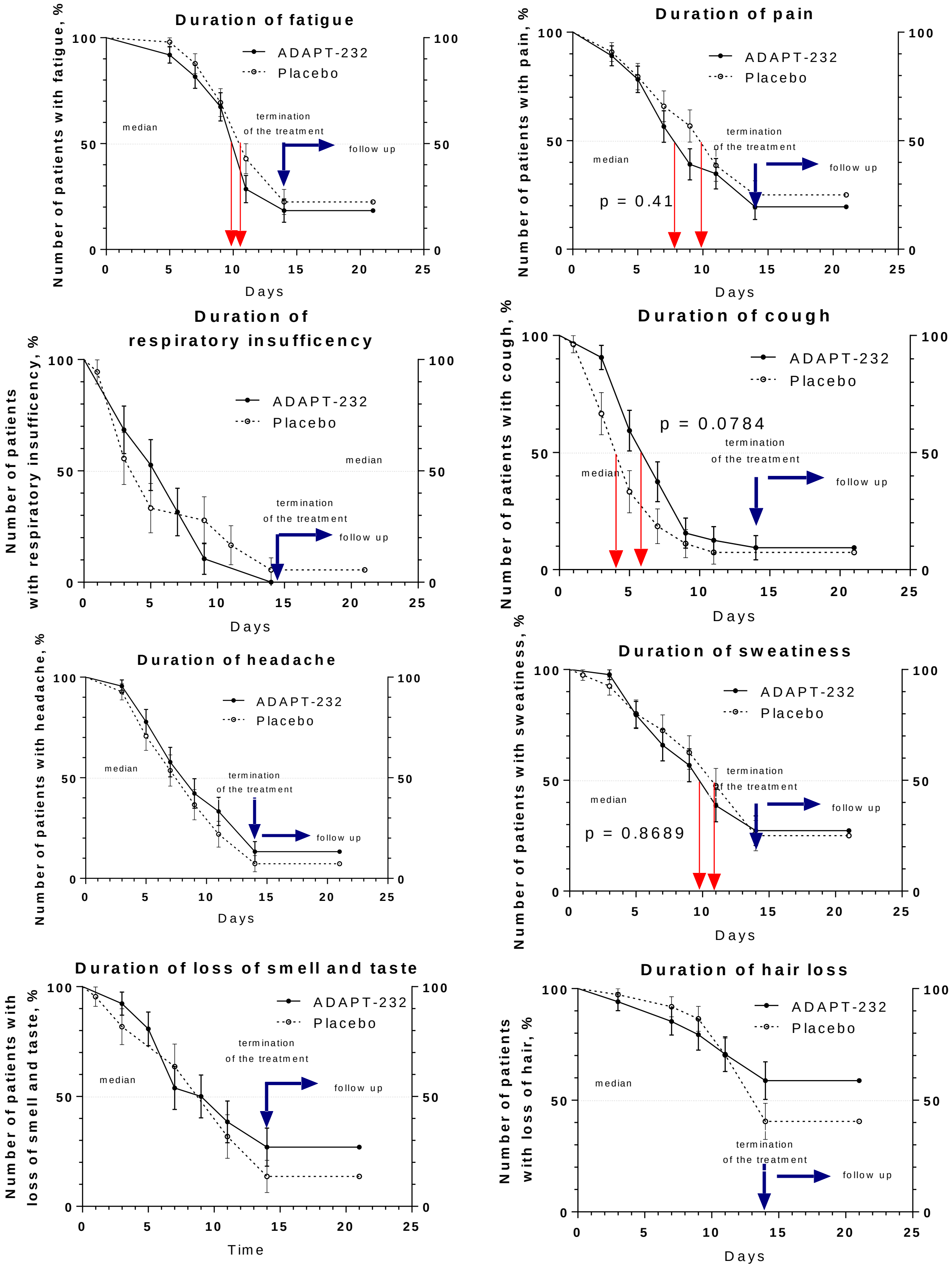

Figure S14. Kaplan-Meier curves show the number of participants who experienced the symptoms of Long COVID over the time from randomization (Day 1) to the end of the treatment (Day 14) and followed up for one week (Day 21): (a) - fatigue, (b) - pain, (c) – respiratory insufficiency, (d) – cough, (e) – headache, (f) – sweatiness, (g) – loss of test and smell, and (h) – hair loss.

Data Sets Analyzed

All enrolled and randomly allocated to treatment were included in the intention to treat analysis. Efficacy subset analysis per-protocol (P.P.) was performed for the subset of patients with Long Covid Symptoms and the baseline (visit 1) and completed the study therapy. A per-protocol (P.P.) analysis aims to identify a treatment effect on the symptoms. Therefore, some patients (from the complete analysis set) need to be excluded from the population used for the P.P. analysis (P.P. population), Table S1.

Table S1 Disposition of patients (Source Data: Supplement 1)

	**Group A**	**Group B**	**Total**
Enrolled	50 (100%)	50 (100%)	100 (100%)
Received at least one dose	49 (98%)	50 (100%)	99 (99%)
Completed study treatments	49 (98%)	50 (100%)	99 (99%)
Completed first visit	50 (100%)	50 (100%)	100 (100%)
Completed all visits	49 (98%)	50 (100%)	99 (99%)
Included in ITT analysis:	49 (98%)	50 (100%)	99 (99%)
Included in analysis per-protocol, P.P:			
• Fatigue	49 (98%)	50 (100%)	99 (99%)
• Headache	45 (90%)	41 (82%)	86(86%)
• Respiration problems	19 (38%)	19 (38%)	38(38%)
• Loss of smell and taste	26 (52%)	**22** (44%)	48(48%)
• Hair loss	33 (66%)	**37** (74%)	70(70%)
• Cough	32 (64%)	**27** (54%)	59(59%)
• Pain in muscles, chest, and joints	46 (92%)	**44** (88%)	90(90%)
• Excess perspiration (sweatiness)	44 (88%)	**41** (82%)	82(82%)
• Stay at home / sick-listed	49 (98%)	**50** (100%)	99(99%)
• Physical activity	49 (98%)	**50** (100%)	99(99%)
• Physical activity (daily walk)	49 (98%)	**50** (100%)	99(99%)
• Decreased attention (d2-test)	49 (98%)	**50** (100%)	99(99%)
• Anxiety	49 (98%)	**50** (100%)	99(99%)
• Depression	49 (98%)	**50** (100%)	99(99%)
• Blood serum cytokines IL-6	49 (98%)	**50** (100%)	99(99%)
• D-Dimer	49 (98%)	**50** (100%)	99(99%)
• C-reactive protein	49 (98%)	**50** (100%)	99(99%)
• Creatinine	49 (98%)	**50** (100%)	99(99%)
