# Supplementary material for: Efficacy of Adaptogens in Patients with Long COVID-19: A Randomized, Quadruple-Blind, Placebo-Controlled Trial
Source: Pharmaceuticals (Basel). 2022 Mar 11;15(3):345. doi: 10.3390/ph15030345 (PMC8953947; doi:10.3390/ph15030345)
Supplement: Supplementary file 1 [file pharmaceuticals-15-00345-s001.zip › pharmaceuticals-1619360-supplementary/Supplement S3 MDA Public Statment Chisan.pdf]

LÄKEMEDELSVERKET
MEDICAL PRODUCTS AGENCY

Läkemedelsverket 2013-05-28

Public Assessment Report
Scientific discussion

Chisan, oral suspension

(*Schisandra chinensis* (Turcz.) Baill., fructus, soft extract (2.0-5.0:1), ethanol 95 %; *Eleutherococcus senticosus* (Rupr. et Maxim) Maxim, radix, soft extract (17-30:1), 1) ethanol 70 %, 2) water; *Rhodiola rosea* L., radix et rhizome, soft extract (2.5-5.0:1), 1) ethanol 70 %, 2) water)

Asp. No: 2006-2004

This module reflects the scientific discussion for the approval of Chisan. The procedure was finalised at 27 November 2008. For information on changes after this date please refer to the module 'Update'.

Postadress/Postal address: P.O. Box 26, SE-751 03 Uppsala, SWEDEN
Besöksadress/Visiting address: Dag Hammarskjölds väg 42, Uppsala
Telefon/Phone: +46 (0)18 17 46 00 Fax: +46 (0)18 54 85 66
Internet: www.mpa.se E-mail: registrator@mpa.se

PDF rendering: Titel 00732530, Version 2.0, Namn Chisan oral suspension OTC ENG PAR

LAY SUMMARY

The Medical Products Agency (Läkemedelsverket) has granted Bringwell AB a traditional-use registration for the herbal medicinal product Chisan, oral suspension. This product is available without prescription and can be bought from pharmacies and other outlets.

Chisan, oral suspension, is traditionally used as an adaptogen in case of reduced performance ability such as tiredness and fatigue.

The active ingredients are soft ethanolic and water extracts from dried roots of eleutherococcus (rysk rot, *Eleutherococcus senticosus* (Rupr. et Maxim) Maxim), dried roots and rhizoma of artic root (rosenrot, *Rhodiola rosea* L.), and ethanolic extract from dried fruit of schisandra (fjärilsranka, *Schisandra chinensis* (Turcz.) Baill.). This registration is based exclusively upon evidence of traditional use as an herbal medicinal product containing extracts of these three plants and not upon data generated from clinical trials. For traditional herbal medicinal products there is no requirement to scientifically prove the effect; adequate evidence of traditional use is sufficient.

The chemical/pharmaceutical quality of the product is acceptable and no new or unexpected safety concerns have been identified during the assessment. It was therefore decided that Chisan could be registered as a traditional herbal medicinal product.

PDF rendering: Titel 00732530, Version 2.0, Namn Chisan oral suspension OTC ENG PAR

I. INTRODUCTION

Bringwell AB has applied for a traditional-use registration for Chisan, oral suspension. The application was submitted under Article 16a traditional use registration for herbal medicinal product of the Directive 2001/83 EC, as amended.

The active substances are:
- *Schisandra chinensis* (Turcz.) Baill., fructus, soft extract (2.0-5.0:1), extraction solvent: ethanol 95 %
- *Eleutherococcus senticosus* (Rupr. et Maxim) Maxim, radix, soft extract (17-30:1), extraction solvent: 1) ethanol 70 %, 2) water
- *Rhodiola rosea* L., radix et rhizome, soft extract (2.5-5.0:1), extraction solvent: 1) ethanol 70 %, 2) water

For approved indications, see the Summary of Product Characteristics.

II. QUALITY ASPECTS

II.1 Introduction

Chisan is presented in the form of oral suspension. 1 ml of oral suspension contains:
- 10 mg soft extract of *Schisandra chinensis* (Turcz.) Baill., fructus (2.0-5.0:1), Extraction solvent: ethanol 95 %, which corresponds to 20-50 mg of dried plant material.
- 2.6 mg soft extract of *Eleutherococcus senticosus* (Rupr. et Maxim) Maxim, radix (17-30:1), Extraction solvent: 1) ethanol 70 %, 2) water, which corresponds to 44-78 mg of dried plant material.
- 3 mg soft extract of *Rhodiola rosea* L., radix et rhizome (2.5-5.0:1), Extraction solvent: 1) ethanol 70 %, 2) water, which corresponds to 7.5-15 mg of dried plant material.

The excipients are: purified water, dark syrup, anhydrous ethanol, glycerol, caramel aroma, polysorbate 80, methyl parahydroxybenzoate (E218), anhydrous citric acid, rosemary extract, propyl parahydroxybenzoate (E216), ginger extract, and potassium sorbate.

The oral suspension is filled into brown glass bottles.

All manufacturers involved in the production operate in accordance with EU-GMP, or where relevant, GACP (Good Manufacturing Practice, respectively Good Agricultural and Collection Practice).

II.2 Drug Substance

The herbal substances are dried root of *Rhodiola rosea* L., dried fruit of *Schisandra chinensis* (Turcz.) Baillon, and dried root of *Eleutherococcus senticosus* (Rupr. et Maxim.) Maxim. The Swedish names of the plants are rosenrot, fjärilsranka and rysk rot, respectively. The specifications of the drug substances are internal but when available very similar to the published herbal monograph in the European Pharmacopoeia.

The extracts are manufactured by Svenska Örtmedicinska Institutet, Gothenburg. The extraction is performed as two extraction cycles, first with 70 % ethanol then with water for *Rhodiola rosea* and *Eleutherococcus senticosus* and twice with 95 % ethanol for *Schisandra*

PDF rendering: Titel 00732530, Version 2.0, Namn Chisan oral suspension OTC ENG PAR

chinensis. The active substance specifications include relevant tests and the limits for impurities have been justified. The content of the markers salidroside, schisandrin and eleutherosides B+E is assayed. Stability studies under ICH conditions have been conducted and the data provided are sufficient to confirm the retest period.

II.3 Medicinal Product

Chisan, oral suspension, is formulated using excipients described in the current European Pharmacopoeia, except for the sweetener (dark syrup) and flavourings (caramel aroma, rosemary and ginger extract) which is controlled according to acceptable internal specifications. All raw materials used in the product are safe with view to possible TSE/BSE risk.

The manufacturing process has been sufficiently described and critical steps identified. Results from the process validation studies confirm that the process is under control and ensure both batch to batch reproducibility and compliance with the product specification.

The tests and limits in the specification are considered appropriate to control the quality of the finished product in relation to its intended purpose.

Stability studies under ICH conditions have been performed and data presented support the shelf life claimed in the SPC.

III. NON-CLINICAL AND CLINICAL ASPECTS

III.1 Introduction

The applicant has presented evidence that *Rhodiola rosea, Schisandra chinensis* and *Eleutherococcus senticosus* individually and in combination, have been used traditionally in the former USSR as “adaptogens”. Preparations containing the combinations of *Rhodiola rosea, Schisandra chinensis* and/or *Eleutherococcus senticosus* have been sold on the Swedish market since the middle of the 1970s.

The Committee on Herbal Medicinal Products (HMPC) of the European Medicines Agency (EMA) has issued a Community monograph on *Eleutherococcus senticosus* radix in 2008 and on *Rhodiola rosea* rhizoma et radix in 2012. There is no Community monograph for *Schisandra chinensis* or the combination of the three active substances. As Chisan was registered in 2008, the Community monographs were not taken into consideration in the national assessment.

III.2 Non-clinical aspects

The applicant has collected available information from the literature in the areas of non-clinical pharmacology and toxicology. This information has been assessed by the MPA and no signals of non-clinical safety concern have been identified. The exact mechanism of action of Chisan, oral suspension, in relation to its traditional medicinal use cannot be considered clarified.

A product/extract specific study on mutagenic activity has been performed. The extract has been shown not to be mutagenic in Ames test.

PDF rendering: Titel 00732530, Version 2.0, Namn Chisan oral suspension OTC ENG PAR

Based on the non-clinical information, both from the literature and the product specific study, no signals of safety concern have been identified that would preclude approval of the ethanolic/water extracts of *Rhodiola rosea* root, *Schisandra chinensis* fruit and *Eleutherococcus senticosus* root as active ingredients in a traditional herbal medicinal product.

III.3 Ecotoxicity/environmental risk assessment

Chisan, oral suspension, is a traditional herbal medicinal product. According to "Guideline on the environmental risk assessment of medicinal products for human use" (EMEA/CHMP/SWP/4447/00), (traditional) herbal medicinal products are exempted from the obligation to present an environmental risk assessment due to the nature of their constituents.

III.4 Clinical aspects

Results of clinical trials concerning clinical efficacy and safety are not required for the registration of a traditional herbal medicinal product.

III.5 Traditional use

The applicant has provided a bibliographic review and expert evidence which show sufficient evidence for the medicinal use of *Rhodiola rosea* root and rhizome, *Schisandra chinensis* fruit and *Eleutherococcus senticosus*, root, alone and in combination, throughout a period of at least 30 years, including at least 15 years within the Community.

With respect to the crude drug, the daily dose (15 ml × 1-2) of 450 mg of *Rhodiola rosea* root, 1200 mg of *Schisandra chinensis* fruit and 2340 mg of *Eleutherococcus senticosus* root is in the same range as the doses given in reference handbooks.

III.6 Clinical safety

The applicant has presented information regarding clinical safety from several handbooks, clinical trials and post marketing experience. Irritability, insomnia and head ache were reported. These adverse effects are reflected in the SPC. The sales figures reported for Chisan, oral suspension, are extensive but no signals of safety concern have been identified through the spontaneous case reports or case reports in the literature.

As no data on use in children are available, Chisan, oral suspension, cannot be recommended for use in children below the age of 12 years.

Due to lack of safety data, the use of Chisan, oral suspension, during pregnancy and lactation is not recommended.

Based on the clinical safety information available, no objections are raised to the approval of Chisan, oral suspension, as a traditional herbal medicinal product.

IV. PRODUCT INFORMATION

PDF rendering: Titel 00732530, Version 2.0, Namn Chisan oral suspension OTC ENG PAR

The product information (Summary of Product Characteristics, Package Leaflet and labelling) has been assessed and accepted by the Medical Products Agency.

V. OVERALL CONCLUSION, RISK ASSESSMENT AND RECOMMENDATION

For Chisan, oral suspension, the handling, manufacture and quality control of raw materials, active substance and finished product are in line with GMP and pharmacopoeial requirements. The applicant has shown that the chemical/pharmaceutical quality is acceptable and can confirm that the process is under control and ensures both batch reproducibility and compliance with the product specification.

There is sufficient evidence for the medicinal use of *Rhodiola rosea* root and rhizome, *Schisandra chinensis* fruit and *Eleutherococcus senticosus* root, alone and in combination, throughout a period of at least 30 years, including at least 15 years within the Community.

No signals of preclinical or clinical safety concern have been identified under normal conditions of use.

Chisan, oral suspension, can be recommended for registration as a traditional herbal medicinal product.

VI. APPROVAL

Chisan, oral suspension, was approved in the national procedure on 2008-11-27.

PDF rendering: Titel 00732530, Version 2.0, Namn Chisan oral suspension OTC ENG PAR

Public Assessment Report – Update

Scope	Procedure number	Product Information affected	Date of start of the procedure	Date of end of procedure	Approval/ non approval	Assessment report attached
						Y/N (version)

Postadress/Postal address: P.O. Box 26, SE-751 03 Uppsala, SWEDEN
Besöksadress/Visiting address: Dag Hammarskjölds väg 42, Uppsala
Telefon/Phone: +46 (0)18 17 46 00 Fax: +46 (0)18 54 85 66
Internet: www.mpa.se E-mail: registrator@mpa.se

PDF rendering: Titel 00732530, Version 2.0, Namn Chisan oral suspension OTC ENG PAR
